# Shear Strength of Exterior Plywood Panels Pressed at Low Temperature

**DOI:** 10.3390/ma2030876

**Published:** 2009-08-04

**Authors:** Pavlo Bekhta, Salim Hiziroglu, Olga Potapova, Jan Sedliacik

**Affiliations:** 1 National University of Forestry and Wood Technology of Ukraine, Department of Wood Based Composites, Lviv, Ukraine; E-Mail: p_bekhta@ukr.net (P.B.); 2 Department of Natural Resources Ecology & Management, Oklahoma State University, Stillwater, Oklahoma 74078-6013, USA; 3 Technical University in Zvolen, Department of Furniture and Wood Materials, Slovakia; E-Mail: janos@vsld.tuzvo.sk (J.S.)

**Keywords:** plywood, veneer, phenolic resin, low press temperature

## Abstract

Plywood manufactured from thin veneer sheets of different species is one of the most traditional structural composite panels. The objective of this study was to produce experimental plywood panels using a temperature of 100 °C, which is 10 to 30% lower than typical press temperature of plywood manufacture. It was determined that shear strength characteristics of the samples were not adversely influenced as function of reduced press temperature. This process can be considered as a promising way to save substantial amounts of energy during pressing processes, which results in a major reduction in overall production costs.

## 1. Introduction

Glueline quality is one of the most important properties of plywood, influencing almost all its physical and mechanical characteristics. Surface quality of the veneer, species, type of adhesive, press time, and press temperature are some of the raw material and manufacturing parameters that need to be taken into consideration to obtain satisfactory plywood shear strengths. In particular press temperature plays an important role in plywood shear strength. In general 110-130 °C is a typical press temperature for panels bonded with phenol formaldehyde adhesive. It is a fact that increased press temperature expedites the polycondensation reaction when adhesive is applied in either liquid or powder form to the surface of veneer [1]. However, in various previous studies it was found that if press temperatures above 130 °C are used for plywood manufacture, significant reduction of cohesive glueline strength takes place, resulting in defective plywood [2,3,4,5,6,7]. Another disadvantage of elevated press temperatures when combined with a high veneer moisture content is having excessive adhesive penetration into veneer as a result of the significant compression coefficient of plywood [1,8]. According to several past studies, it was concluded that the pressure of gas-vapor mixture in glueline is increased with increasing press temperature, causing development of bubbles within the glueline [1,9,10,]. The amount of such bubbles can increase to 10% in the case of press temperatures ranging from 118 to 120 °C, but the development of these bubbles decreases by several percent as the press temperature is reduced to 100-115 °C [11]. Based on another previous work it was found that vapour and gas conductivity of hardwood is higher than that of softwood [10]. Therefore, it appears that birch and other hardwood veneers could possibly be pressed at higher temperatures. In general, using press temperatures below 100 °C is not recommended due to its adverse effect on the glueline curing time [12,13]. However, some other studies have revealed that using a lower temperature in the plywood manufacture press line had no influence if phenolic and resorcinol adhesives, combined with different hardeners including dichrome sodium, are used [14,15,16,17,18]. Orlov also used different combination of urea formaldehyde resin and to successfully press plywood samples at a temperature of 100 °C [17]. Dispersed silica powder was added as active filler into the adhesive to press softwood veneer sheets at a temperature of 105-110 °C without any problems in a past experimental work [15]. Pressing plywood at low temperature possibly does not cause adverse effects on panel properties due to superflows of vapor-gas pressure developed within the glueline [19,20,21]. Cold pressing can also be considered an alternative method, but low productivity is its main shortcoming. Therefore, phenolic resins along with hardeners would be considered as an ideal process and this eliminates problems faced when high press temperatures are used. The overall objective of this study was to evaluate the shear strength properties of plywood panels produced experimentally using a low press temperature of 100 °C.

## 2. Materials and Methods

Commercially manufactured birch (*Betula pubescens*) and beech (*Fagus orientalis* L.) veneer sheets were used for the experiments. Three-layer 300 mm by 300 mm panels were manufactured from two different species and their combinations. The birch and beech veneer samples had thicknesses of 1.5 and 1.8 m, respectively.

Phenol formaldehyde resin modified with resorcinol, alkylresorcinol, and melamine was used as binder at a spread rate of 140 g/m^2^. Table 1 displays the adhesive and additives compositions used for the experiments. A type-RV2 rotary viscosimeter and a Hanna H1 22 pH meter were employed to determine the dynamic viscosity and pH of the adhesive, respectively. Setting time of the adhesive was also measured at three temperatures, namely 100 °C, 120 °C and 150 °C. Specifications of adhesive mixtures are shown in Table 2. Plywood samples were manufactured using a computer controlled press at a temperature of 100 °C and a pressure of 1.8 MPa for 6 min. Control samples were also produced at the same pressure and press time using two press temperatures of 120 and 150 °C. Shear stress samples were cut from each panels based on European Standard number 314 after they were conditioned in controlled climate chamber at a temperature of 20 °C and a relative humidity of 65% for two weeks [22].

**Table 1:** Adhesive compositions.

	Adhesive Mixtures								
Adhesive components	0	1	2	3	4	5	6	7	8
Phenol Formaldehyde	100	100	100	100	100	100	100	100	100
Alkylresorcinol	-	3	3	-	-	-	-	-	-
Resorcinol	-	-	-	3	3	3	2	3	3
Paraformaldehyde	-	7	7	6	6	10	5	5	5
Hydrogen peroxide	-	-	-	-	1	1	1	1	1
Combined hardener:	-	10	15	13	13	10	7	5	7
Melamine	-	-	-	-	-	6	-	-	-
Urea	-	24	7	6	6	-	3	3	3
Dichromate ammonium	-	-	-	6	6	-	-	-	-
Dichromate potassium	-	24	7	-	-	6	-	-	-
Dichromate sodium	-	-	-	-	-	-	3	3	3

**Table 2:** Properties of the adhesives mixtures.

Properties of adhesive type	Adhesive mix types								
	0	1	2	3	4	5	6	7	8
Dynamic viscosity (mPa)	658	776	922	1,800	2,020	1,342	1,293	732	849
Assembly life (min)	400	90	84	55	51	65	69	79	76
pH	11.42	10.98	11.06	10.76	10.42	10.93	10.96	10.88	10.92
Setting time (s)									
100 °С	202	124	133	107	98	107	118	121	130
120 °С	67	32	26	37	30	25	48	49	50
150 °С	-	25	26	30	22	22	39	34	34

## 3. Results and Discussion

Table 3 lists the shear strength values of plywood samples manufactured under different conditions. The strength values of the samples made from birch using only phenol formaldehyde resin without any additives increased with increasing press temperature. Samples pressed at a temperature of 150 °C had an average shear strength value of 2.31 MPa, which was 7.21 times higher than those of those pressed at a temperature of 120 °C. Overall, panels manufactured using 1% hydrogen peroxide resulted in slightly lower shear strength than those of rest of the samples. This could be related to the influence of hydrogen peroxide on adhesive properties. It was determined that adhesive mixed with 1% hydrogen peroxide had reduced setting time, shelf life and increased viscosity by 8.4%, 7.3%, and 12.2%, respectively. The significant decrease of shear strength of these samples could be due to the above modification in adhesive chemistry. It seems that all panels manufactured using 1% hydrogen pressed at 100 °C temperature had similar shear strength values to each other. These values were higher than those of birch samples which were pressed at a temperature of 100 °C. Samples made with adhesive having 3% dichromate ammonium as hardener did not show any noticeable difference in their shear strength properties from each other, as can be seen in Table 3. When mixtures of hardeners ranging from 5% to 15% by weight of the adhesive were used to manufacture all three types plywood samples, none of these exhibited any superiority in their shear strength values over the others. Overall, birch samples showed higher shear strength those of made from beech and combination of beech and birch for all types of additives and resin mixtures. This is possibly due to anatomical structure of birch, with more diffuse pores and having higher density than that of beech. According to European Standard EN 314, the minimum shear strength requirement for exterior plywood is 1.0 MPa and based on the preliminary test results, all samples satisfied this limit [18]. It appears that using a 100 °C press temperature to manufacture experimental panels from birch, beech, or combinations of two did not adversely influence their shear strength. Panels made with only phenol formaldehyde adhesive not containing any other additives and pressed at a temperature of 140 °C had 1.83 MPa share strength, which was lower than that of 12 types of panels out of 24. Overall ratio based on the results is 50% and this ratio increased to 75% when the shear strength of the above samples is compared to that of those pressed at a temperature of 130 °C. It is clear that even with a reduction in press temperature by 30% or 40%, the overall energy cost savings throughout the production sequence could be substantial. Regardless of the influence of the additives used in that adhesive and the effect of different species, such saving, would not only enhance the sustainability of resources but also provide manufactured plywood panels with acceptable strength properties

**Table 3:** Shear strength of the samples.

Adhesive type	Press temperature, (°C)	Panel type	Shear strength (MPa)
0	100	Birch	-
	120		0.32 (0.12)
	130		1.65 (0.67)
	140		1.83 (0.67)
	150		2.31 (0.34)
1	100	Birch	2.42 (0.54)
		Beech	1.57 (0.25)
		Birch-Beech	1.50 (0.24)
2	100	Birch	2.45 (0.40)
		Beech	1.52 (0.45)
		Birch-Beech	1.47 (0.33)
3	100	Birch	1.87 (0.42)
		Beech	1.58 (0.33)
		Birch-Beech	0.78 (0.68)
4	100	Birch	1.85 (0.42)
		Beech	1.34 (0.31)
		Birch-Beech	0.99 (0.39)
5	100	Birch	2.15 (0.47)
		Beech	2.26 (0.29)
		Birch-Beech	1.49 (0.21)
6	100	Birch	2.37 (0.44)
		Beech	2.19 (0.21)
		Birch-Beech	1.35 (0.28)
7	100	Birch	2.15 (0.46)
		Beech	2.18 (0.29)
		Birch-Beech	1,73 (0.35)
8	100	Birch	2.66 (0.46)
		Beech	1.98 (0.31)
		Birch-Beech	1,74 (0.35)

## 4. Conclusions

In conclusion, it was found that the shear strength characteristics of the plywood samples tested in this work were not adversely influenced by a reduction of the press temperature. It appears that using hydrogen peroxide reduced setting time and shelf life of the adhesive. However hardeners used for panel manufacture did not show any significant impact on the shear strength of the samples. In further studies, it would be desirable to examine panels made from different species using different types of adhesive content to obtain more comprehensive information on the properties of such plywood panels.
